# Virulence Factor Genes Detected in the Complete Genome Sequence of *Corynebacterium uterequi* DSM 45634, Isolated from the Uterus of a Maiden Mare

**DOI:** 10.1128/genomeA.00783-15

**Published:** 2015-07-30

**Authors:** Christian Rückert, Martin Kriete, Sebastian Jaenicke, Anika Winkler, Andreas Tauch

**Affiliations:** a Department of Biology, Massachusetts Institute of Technology (MIT), Cambridge, Massachusetts, USA; b Institut für Genomforschung und Systembiologie, Centrum für Biotechnologie (CeBiTec), Universität Bielefeld, Bielefeld, Germany; c Bioinformatics Resource Facility (BRF), Centrum für Biotechnologie (CeBiTec), Universität Bielefeld, Bielefeld, Germany

## Abstract

The complete genome sequence of the type strain *Corynebacterium uterequi* DSM 45634 from an equine urogenital tract specimen comprises 2,419,437 bp and 2,163 protein-coding genes. Candidate virulence factors are homologs of DIP0733, DIP1281, and DIP1621 from *Corynebacterium diphtheriae* and of sialidase precursors from *Trueperella pyogenes* and *Chlamydia trachomatis*.

## GENOME ANNOUNCEMENT

*Corynebacterium uterequi* was proposed as a novel nonlipophilic species of the genus *Corynebacterium* by Hoyles et al. in 2013 (1). The description of this species is based on molecular and phenotypic data of three isolates associated with the urogenital tract of mares in Scotland and Sweden. Bacteria with similar morphological appearance were detected in additional uterus samples from mares with clinical problems and from mares serving as routine controls. This finding suggests that *C. uterequi* represents a commensal bacterium of the equine urogenital tract (1). The type strain *C. uterequi* DSM 45634 (VM 2298^T^) was isolated in pure culture from a maiden mare suffering from an increased amount of liquid in the uterus. The 16S rRNA gene sequence of the type strain shared 95.01% sequence similarity with the nearest taxonomic relative *Corynebacterium lubricantis* that was isolated from a coolant lubricant in Germany (1, 2). Here, we present the complete genome sequence of the type strain *C. uterequi* DSM 45634 to identify candidate virulence factor genes (3–6).

Genomic DNA of *C. uterequi* DSM 45634 was obtained from the Leibniz Institute DSMZ. The genomic DNA was sequenced in a combined approach using a whole-genome shotgun library and a mate pair library. The whole-genome shotgun library was constructed with the Nextera DNA sample preparation kit (Illumina) and was sequenced in a paired-end run using the MiSeq reagent kit v2 (500 cycles) and the MiSeq desktop sequencer (Illumina). This shotgun sequencing resulted in 1,569,676 paired reads and 253,713,569 detected bases. The paired reads were assembled with the Roche GS De Novo Assembler software (Newbler; release 2.8), yielding 22 scaffolds with 38 scaffolded contigs. A 7-kb mate pair library was prepared with the Nextera mate pair sample preparation kit according to the gel-plus protocol. The mate pair library was sequenced with the MiSeq reagent kit v3 (600 cycles) and 459,853 mate pair reads were added to the initial Newbler assembly. The gap closure step was facilitated by the Consed software (version 26) (7). Gene prediction was performed with the Prodigal software (8) and the functional annotation of the detected protein-coding regions was supported by the IMG/ER pipeline (9).

The chromosome of *C. uterequi* DSM 45634 has a size of 2,419,437 bp with a mean G+C content of 65.5%. The automatic annotation of the genome sequence revealed 2,163 protein-coding regions, 53 tRNA genes, and 5 rRNA operons. A BLASTp search for potential virulence factors (3–6) led to the detection of homologs of DIP0733, a multifunctional virulence factor of *Corynebacterium diphtheriae* (10, 11), DIP1281, involved in adhesion and internalization of *C. diphtheriae* (12), and DIP1621, an adherence factor of *C. diphtheriae* (13). Additional candidate virulence factors are lipases of the LIP family (14), a secreted SGNH hydrolase (14), and sialidases with similarity to proteins from *Chlamydia trachomatis* and *Trueperella pyogenes* (15). At least the predicted sialidases of *C. uterequi* DSM 45634 represent niche factors (16), showing the adaptation of this bacterium to a distinct equine environment that is rich in glycoconjugates during pregnancy (17).

### Nucleotide sequence accession number.

This genome project has been deposited in the GenBank database under the accession no. CP011546.
